# Development and external validation of a clinical prediction model for predicting quality of recovery up to 1 week after surgery

**DOI:** 10.1038/s41598-023-50518-1

**Published:** 2024-01-03

**Authors:** Stefan van Beek, Daan Nieboer, Markus Klimek, Robert Jan Stolker, Hendrik-Jan Mijderwijk

**Affiliations:** 1 Department of Anaesthesiology, Erasmus University Medical Centre, Dr. Molewaterplein 40, 3015 GD Rotterdam, The Netherlands; 2 Department of Public Health, Erasmus University Medical Centre, Rotterdam, The Netherlands; 3 Department of Neurosurgery, Medical Faculty, Heinrich Heine University, Düsseldorf, Germany

**Keywords:** Health care, Quality of life, Outcomes research, Epidemiology

## Abstract

The Quality of Recovery Score-40 (QoR-40) has been increasingly used for assessing recovery after patients undergoing surgery. However, a prediction model estimating quality of recovery is lacking. The aim of the present study was to develop and externally validate a clinical prediction model that predicts quality of recovery up to one week after surgery. The modelling procedure consisted of two models of increasing complexity (basic and full model). To assess the internal validity of the developed model, bootstrapping (1000 times) was applied. At external validation, the model performance was evaluated according to measures for overall model performance (explained variance (*R*^2^)) and calibration (calibration plot and slope). The full model consisted of age, sex, previous surgery, BMI, ASA classification, duration of surgery, HADS and preoperative QoR-40 score. At model development, the *R*^2^ of the full model was 0.24. At external validation the *R*^2^ dropped as expected. The calibration analysis showed that the QoR-40 predictions provided by the developed prediction models are reliable. The presented models can be used as a starting point for future updating in prediction studies. When the predictive performance is improved it could be implemented clinically in the future.

## Introduction

Patient reported outcomes measures (PROMs) as quality indicator after medical interventions are of growing interest^1^. Measuring health status after anaesthesia and surgery is commonly evaluated by the QoR-40, a quality of recovery (QoR) scoring system. The QoR-40 questionnaire is a validated PROM that is able to quantify the postoperative health status of a patient up to one week postoperatively^2^. QoR-40 has been increasingly used for assessing recovery after patients undergoing major surgical procedures but also in patients undergoing minor surgical procedures performed on a day-case base^3–5^. Furthermore, the QoR-40 is a patient-friendly tool that is available in multiple languages and provides a valid broad assessment of recovery after anaesthesia and surgery^6^. Lower QoR-40 scores results in increased length of hospital stay (LOS), lower patient satisfaction and possible extra medical costs^5^.

Therefore, accurate identification of patients at risk of poor quality of postoperative recovery is vital, not only to inform patients and their relatives on their anticipated postoperative recovery, but also to have the possibility to initiate treatments for these patients.

Clinical prediction models are powerful tools that aim to assist clinicians in providing evidence-based material to facilitate decision-making. A robustly developed and validated clinical prediction model could help clinicians identifying patients at risk of poor recovery. Consequently, interventions could be designed and performed to improve the quality of recovery of these patients. Recently a pre-operative rehabilitation recovery protocol showed improved quality of recovery^7^. Also, more patient-tailored care could be organized for patients with the need for additional care or an extended length of stay in the hospital.

To date, a prediction model that predicts quality of recovery after major surgery and minor surgery is lacking. The aim of the present study was to develop and externally validate a clinical prediction model that predicts quality of recovery up to one week after surgery. This clinical prediction model can be updated in future research and serve as a clinical tool for perioperative decision making.

## Methods

This study used two datasets from existing randomised controlled trials (RCT)^3,4^. The Medical Ethical Board of the Erasmus Medical Centre and the Netherlands Central Committee on Research involving Human Subjects (CCMO) approved both studies, the first on 23 August 2010 (with ClinicalTrials.gov identifier: NCT01441843) and the second on 18 June 2013 (with ClinicalTrials.gov identifier NCT01993459). Both studies were performed in accordance with relevant guidelines and regulations. All patients included in both RCTs have provided written informed consent prior to any data collection.

### Model development set

Data from a double-blinded RCT evaluating the effect of premedication with a benzodiazepine on quality of recovery in patients undergoing elective (major) inpatient surgery were used to develop the prediction model^4^. This RCT included 192 adult patients (aged at least 18 years) from July 2014 until September 2015 who were admitted for laparotomy for abdominal pathologies. QoR-40 was assessed on POD (postoperative day) 0 and POD 7. The detailed study methodology and the CONSORT diagram have been published elsewhere^4^.

### Model validation set

To assess the model’s performance in an external population, data from another RCT evaluating the effect of premedication with a benzodiazepine on quality of recovery conducted in a day-case (minor) surgery population was used (N = 398), recruited in 2010 and 2011^3^. The study design and methodology were comparable to the RCT of which patient data were used for model development. The detailed study methodology and the CONSORT diagram are available elsewhere^3^.

### Outcome definition

A clinical prediction model was developed to predict quality of recovery one week after surgery. The QoR-40 score was used as a measurement for quality of recovery.

The QoR-40 contains five scales assessing physical comfort, emotional state, physical independence, psychological support and pain. Each item within the scales is scored on a five-point Likert scale, and the QoR-40 score is calculated as the total sum of scores. The higher the score the higher the quality of recovery, ranging from a minimum score of 40 and a maximum score of 200. A difference of 6.3 points or more between measurements is considered a clinically relevant difference^8^.

In preoperative patients, scores of QoR-40 are generally between 150 and 190 and patients undergoing minor, intermediate, and major surgery have mean ± SD postoperative QoR-40 scores of 178 ± 17, 173 ± 17, and 166 ± 15, respectively^9^. The QoR-40 questionnaire has good psychometric properties with good validity, test–retest reliability and internal consistency (Cronbach’s α = 0.93). The questionnaire is easy to use with a good clinical acceptability^10^. It takes approximately 7 min to complete and it can easily be performed by the patient without help of medical personnel.

### Candidate prognostic variables

Based on subject matter knowledge and a systematic literature review on Pubmed using the search term ((“Postoperative Period”[Mesh]) AND “Anesthesia Recovery Period”[Mesh] AND “quality of recovery”) selecting all articles with full text availability until 31-12-2021. We have included possible predictors from the literature describing the quality of recovery in patients after surgery including all types of anaesthesia. We identified the following candidate prognostic variables: age, sex, having previous surgery, body mass index (BMI), ASA-Classification, duration of the surgical procedure, the Hospital anxiety and Depression Scale (HADS) and QoR-40 at baseline^5,10–13^.

The HADS measures negative moods and consists of two seven-item scales, one measuring depression and measuring anxiety. A higher score indicates higher degrees of negative moods with a score range from 0 to 42. For Dutch population HADS has an internal consistency of 0.88 (Cronbach’s α)^14^.

All candidate prognostic variables were assessed prior to the surgical procedure on the day of surgery (POD 0). The treatment allocation (benzodiazepine versus placebo) as randomised in both RCTs was included in the analysis to control for possible confounding.

### Sample size considerations

Complete case analysis was performed because missingness on the outcome and candidate prognostic variables was low^15,16^. The model development data set included 174 complete cases (of 192 included cases). The external validation set included 381 complete cases (of 398 included cases). The effective sample size, using the rule of thumb using minimal 10 events per variable, for the development and external validation was adequate^17^. However to calculate the sample size we know that the minimal value of n should meet the following four key criteria: (i) small optimism in predictor effect estimates as defined by a global shrinkage factor of ≥ 0.9; (ii) small absolute difference of ≤ 0.05 in the apparent and adjusted R2 ; (iii) precise estimation of the model's residual standard deviation; and similarly, (iv) precise estimation of the mean predicted outcome value (model intercept). The criteria require prespecification of the user's chosen p and the model's anticipated R2 as informed by previous studies^18^.

Following the guidelines set out for the sample size calculations, we assumed that the expected R^2^ would be 0.5, as we include a baseline measurement of the outcome in our prediction model. For criteria (iii) and (iv) we calculated the MMOE and observed that the expected MMOE was equal to 1.12 which we judge sufficiently close to the recommended cut-off value in the paper to proceed with the model development.

### Model development, performance, validation and presentation

The model generation was in accordance to recent methodology^15,17^. The TRIPOD-guidelines were adhered to^19^. BM® SPSS® Statistic and R were used as statistical software.

#### Model development

Continuous variables were kept continuous to avoid loss of prognostic information. We assessed the assumptions of the linear regression model, such as normality of the residuals, possible non-linear associations between continuous variables and outcome, and possible influential measurements graphically. We detected no major violations of the underlying assumptions. Among the candidate predictor variables, only QoR-40 as assessed at baseline seems to have some non-linearity in the association with QoR-40 after 1 week. In detail, the association was linear until the score of 180 but then stayed relatively constant. Consequently, for QoR-40 at baseline we used piecewise linear functions which rises until 180 and then remains constant. ASA-Classification 3 and 4 were collapsed since only 3 patients were labelled as ASA 4 in the development set.

Using linear regression, a prediction model to predict quality of recovery one week after surgery was developed. The modelling procedure consisted of two models of increasing complexity:

1. Basic model including patient age, sex, having at least one previous operation, BMI, ASA-score, and the duration of the surgical procedure.
2. Full model that extended the basic model to include HADS and QoR-40 as assessed prior to the surgical procedure.

#### Model validation

For internal validation, 1000 bootstrap samples were used to indicate the shrinkage factor. The performance of the model—i.e. explained variance and calibration—was evaluated. In the next step, the performance of the identified prediction model was assessed according to calibration (calibration plots and calibration slopes) and overall model performance in the external validation set. The overall model performance was calculated as explained variance (R^2^), which measures the proportion of the variability in the quality of recovery score that is explained by the prediction model. The R^2^ ranges from 0 to 100%. Calibration was assessed for both models with a calibration plot and corresponding slope and calibration-in-the-large^20^. Here, the predicted QoR-40 score is plotted against the observed QoR-40 score to show similarities between the predictions of the model with the observed outcomes. A loess smoother was used to estimate the association between predicted and observed values of the QoR-40 score. The 45-degree line indicates perfect agreement. The calibration slope measures whether predictor effects are on average too extreme or too conservative and should ideally be equal to 1. The calibration slope is estimated by fitting a linear regression model using the linear predictor of the prediction model as only covariate. The calibration-in-the-large quantifies if predictions made by the model are on average correct and is estimated by fitting a linear regression model using the linear predictor as an offset variable. Ideally the calibration-in-the-large should be equal to zero. A positive value indicates on average underestimation of the QoR-40 score, while negative values indicate overestimation of the QoR-40 score.

#### Model presentation

The equation of the constructed prediction model will be presented in the appendix, making external validation by independent researchers possible. For prognostication, i.e. using the prediction model for the calculation of QoR-40 in new patients, a nomogram of the model is created.

## Results

### Descriptive analysis

The development data set contained 174 complete cases and the validation data set contained 381 complete cases (Table 1). One week after surgery the QoR-40 score was lower (median 171.5) in the development set compared to the validation set (median 177). The patients in the development set showed higher age, were more likely to have had previous surgery, and had higher BMI scores. Furthermore, as expected, inpatients had longer duration of surgery. The proportion of females was comparable in both sets (37% and 43%). As expected, most cases in the validation set were ASA class 1 or 2 (99%) in contrast to the development set where most cases were ASA class 3 (48%). Both, the HADS score (median 7) and QoR-40 score (median 184) before surgery, were comparable in both sets.

**Table 1:** Patients characteristics from the development and validation data set.

	Development set	Validation set	P*
Patient total	174	381	
QoR-40^a^ POD^b^ 7 median [IQR])	171.50 [158.00, 183.00]	177.00 [159.00, 193.00]	< 0.001
Age (median [IQR])	59.00 [48.00, 67.00]	37.09 [28.92, 49.42]	< 0.001
Sex Female (%)	65 (37.4)	163 (42.8)	0.266
Previous surgery Yes (%)	158 (90.8)	312 (81.9)	0.010
BMI^c^ (median [IQR])	25.29 [23.10, 28.74]	24.61 [22.40, 27.75]	0.027
ASA^d^ n(%)			< 0.001
1	13 (7.5)	245 (64.3)	
2	78 (44.8)	132 (34.6)	
3	83 (47.7)	4 (1.0)	
Duration of surgery^e^ (median [IQR])	178.00 [117.00, 267.00]	41.00 [26.00, 61.00]	< 0.001
HADS^f^ POD 0 (median [IQR])	7.00 [4.00, 10.00]	7.00 [4.00, 10.00]	0.803
QoR-40 POD 0 (median [IQR])	184.00 [172.00, 192.00]	184.00 [164.00, 194.00]	0.790

*Threshold for significance was *P* < 0.05.

^a^*QoR-40* Quality of Recovery 40 Score.

^b^*POD* Postoperative day.

^c^*BMI* body mass index.

^d^*ASA* American Society of Anesthesiologists Classification.

^e^Duration of surgery in minutes.

^f^*HADS* Hospital Anxiety and Depression Scale, *IQR* Interquartile range. Categorical data was tested with Pearson’s chi-squared test, continuous data was tested with student’s t-test or the Mann–Whitney U test for non-normally distributed data.

### Model development and internal validation

Table 2 displays the results of the basic model. Six variables were included in the basic model: age, sex, previous surgery, BMI, ASA classification and duration of surgery. Trends seen in Table 2 and the nomogram were; women, having higher ASA classifications or longer duration of surgery decreased the postoperative QoR-40 score. In contrast, higher age and experience with previous surgery increased the quality of postoperative recovery. Only duration of surgery was statistically significant.

**Table 2:** Model development: results of the basic model.

Variable	Units	Coefficient	CI 95%^e^	P*
(Intercept)		163.03	[142.58;183.49]	< 0.001
Age^a^		1.87	[-0.08;3.82]	0.062
Sex	Male	Ref		
	Female	− 3.79	[− 9.37;1.78]	0.184
Previous surgery	No	Ref		
	Yes	1.54	[− 7.20;10.28]	0.730
BMI^b^		0.40	[− 0.16;0.96]	0.163
ASA^c^	1	Ref		
	2	− 3.53	[− 13.74;6.67]	0.498
	3	− 5.11	[− 15.62;5.39]	0.341
Duration^d^		− 3.15	− -4.56; − 1.73]	< 0.001

*Threshold for significance was *P* < 0.05.

^a^Age/10.

^b^*BMI* body mass index.

^c^*ASA* American Society of Anesthesiologists Classification.

^d^Duration of surgery per hour.

^e^*CI* Confidence interval 95%.

**Figure 1:**
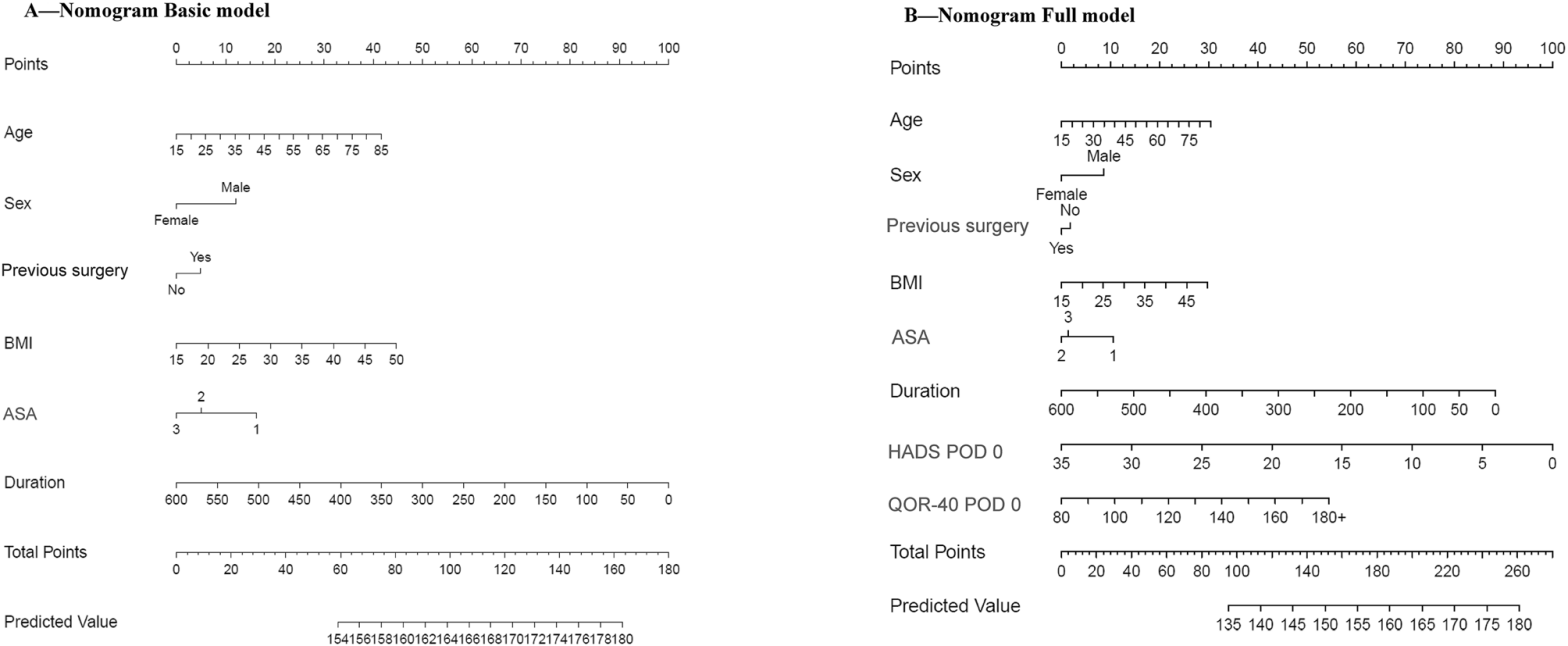
(**A**) Nomogram Basic model. Age in years, BMI = body mass index, *ASA*American Society of Anesthesiologists Classification, Duration of surgery in minutes. The nomogram graphically represents the effect of each predictor on the outcome. Each value of a predictor is assigned to a score (upper scale; Points). The sum of these scores (= total points) corresponds to a predicted value on the lower scale. (**B**) Nomogram Full model. Age in years, *BMI* Body mass index, *ASA* American Society of Anesthesiologists Classification, Duration of surgery in minutes, *HADS* Hospital Anxiety and Depression Scale, *POD* Postoperative day, *QoR-40* Quality of Recovery 40 Score. The nomogram graphically represents the effect of each predictor on the outcome. Each value of a predictor is assigned to a score (upper scale; Points). The sum of these scores (= total points) corresponds to a predicted value on the lower scale. Directions for use: Locate the patient's preoperative QoR-40 on the QoR-40 POD 0 axis. Draw a line straight upward to the point's axis to determine how many points the patient obtains. Redo this for each prognostic variable. Sum the achieved points. Locate the final sum of the points on the Total Points axis. Draw a line straight down to find the patient's expected QoR-40 scores at one week. For example, a male patient (8 points), 60 years old (20 points), who had previous surgery (0 points), with a BMI of 30 (12.5 points), ASA 1 classification (10 points) for a surgery with a duration of 200 min (57.5 points). He scored 150 on the QoR-40 POD0 (37.5 points) and 25 on the HADS POD0 (28 points). This patient has 173.5 total points which corresponds approximately to a predicted QoR40 of 157. Following the appendix formula this patient will have a calculated predicted QoR-40 score of 155.33.

Table 3 shows the results of the full model. The full model contains eight variables adding the preoperative HADS and QoR-40 score to the basic model. HADS and duration of surgery had the highest contribution based on their statistical significance. Trends were as following; patients having a higher preoperative HADS score, a lower baseline QoR-40, longer duration of surgery and having a higher ASA classification are at risk of lower quality of postoperative recovery. Furthermore, females and younger patients with a lower BMI, and having surgical experience before are associated with lower quality of postoperative recovery. This is illustrated in the nomograms (Fig. 1) showing the strength of the association of the predictors for the full model to the predicted quality of recovery value. The nomograms show the probability for the outcome through a score (upper scale; points) for each predictor value. The lower two scales are then used to convert the sum of these scores (total points) to a predicted value on the QoR-40.

**Table 3:** Model development: results of the full model.

Variable	Units	Coefficient	CI 95%^h^	P*
(Intercept)		145.88	[99.95;191.82]	< 0.001
Age^a^		1.35	[− 0.49;3.20]	0.151
Sex	Male	Ref		
	Female	− 2.70	[− 7.95;2.56]	0.316
Previous surgery	No	Ref		
	Yes	− 0.55	[− 8.81;7.71]	0.896
BMI^b^		0.27	[− 0.27;0.80]	0.332
ASA^c^	1	Ref		
	2	− 3.29	[− 12.86;6.28]	0.502
	3	− 2.86	[− 12.76;7.03]	0.571
Duration^d^		− 2.76	[− 4.10;-1.42]	< 0.001
HADS^e^ POD^f^ 0		− 0.89	[− 1.41;-0.37]	0.001
QoR-40^ g^ POD 0		0.17	[− 0.05;0.39]	0.138

*Threshold for significance was *P* < 0.05.

^a^Age/10.

^b^*BMI* Body mass index.

^c^*ASA* American Society of Anesthesiologists Classification.

^d^Duration of surgery per hour.

^e^*HADS* Hospital Anxiety and Depression Scale.

^f^*POD* Postoperative day.

^g^*QoR-40* Quality of Recovery 40 Score.

^h^*CI* Confidence interval 95%.

Internal validation with bootstrapping (1000 samples) suggested that the basic and the full model needed a shrinkage factor of 0.79 and 0.87 respectively because of too large predictor-outcome associations. The *R*^2^ of the basic model was 0.13 (optimism corrected *R*^2^ equalled 0.05). However, the *R*^2^ of the full model was 0.24 (optimism corrected *R*^2^ equalled 0.15).

### External validation

**Figure 2:**
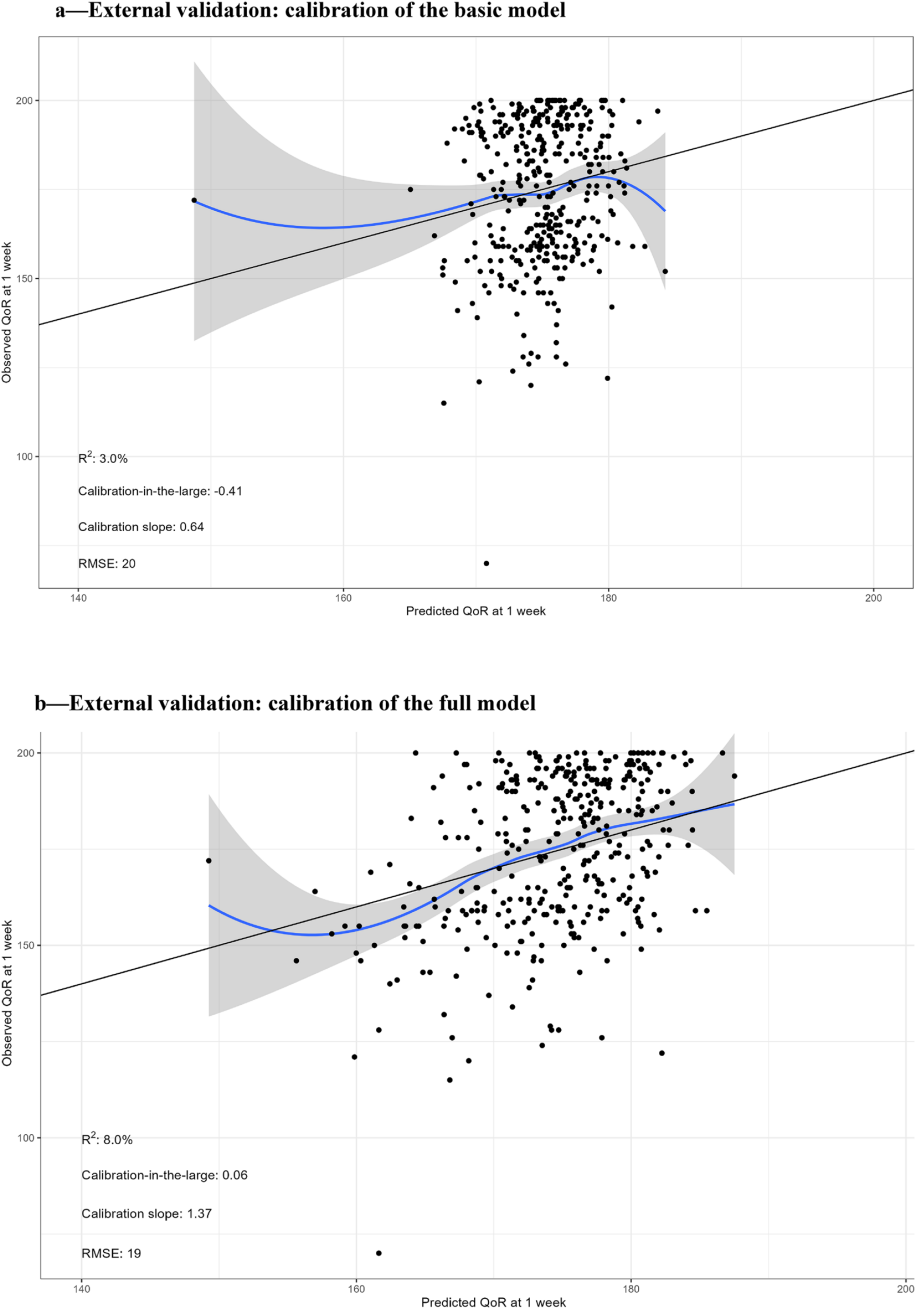
(**a**) External validation: calibration of the basic model. Visualisation of model calibration. The plots show the observed outcome versus the predicted outcomes. The observed QoR-40 scores are shown as black dots. The black (diagonal) line shows the perfect calibration. The blue line shows the predicted QoR-40 score averages. The grey area around the blue line corresponds to the 95% confidence interval. A blue curve close to the diagonal indicates that predicted averages correspond well to average observed response. If the blue curve is further away from the diagonal it means there is an over or underestimation of the average response. (**b**) External validation: calibration of the full model. Visualisation of model calibration. The plots show the observed outcome versus the predicted outcomes. The observed QoR-40 scores are shown as black dots. The black (diagonal) line shows the perfect calibration. The blue line shows the predicted QoR-40 score averages. The grey area around the blue line corresponds to the 95% confidence interval. A blue curve close to the diagonal indicates that predicted averages correspond well to average observed response. If the blue curve is further away from the diagonal it means there is an over or underestimation of the average response.

Calibration is highly consequential to prediction model-based decision making, and, therefore, moderate calibration is desired^17,21^. Moderate calibration implies that estimated risks correspond to observed proportions. The constructed model showed moderate calibration on patients undergoing day-case surgery, as depicted in the calibration curves with 95% confidence intervals (Fig. 2). The calibration plot shows small confidence intervals around the majority of the scores. However, the QoR-40 scores < 160 and > 180 showed large uncertainty. The calibration slope was approximately 1 for the two models: 0.64 for the clinical model, while the slope for the full model was 1.34. Additionally, we calculate the calibration-in-the-large of the developed models which measures whether estimates were on average too low or too high. These were -0.41 and 0.55 for the clinical and full model respectively, indicating a very good overall calibration. The explained variance of *R*^2^ of the full model was 0.08.

Table 4 shows the RMSE (root-mean-squared-error). At external validation the RMSE for the basic model was 20 and for the full model was 19 (see Fig. 2).

**Table 4:** RMSE.

	Basic model	Full model
Optimism corrected RMSE	17	16
RMSE at external validation	20	19

*RMSE* Root-mean-squared-error.

### Model presentation

The nomogram in Fig. 1A,B enables the estimation of QoR-40 one week after surgery. One example patient, to illustrate the use of the prediction model and its nomogram, is presented in the legend. The prognostic equation is attached in the appendix.

## Discussion

We developed and externally validated a clinical prediction model that predicts the quality of recovery score one week after surgery. Our goal was to build a clinical tool that could help identifying patients at risk of poor recovery. The full prediction model contains age, sex, previous surgery, BMI, ASA classification, duration of surgery, HADS and QoR-40 score preoperatively. To our knowledge this is the first prediction model that tries to predict quality of recovery after surgery. We used common available clinical variables to make this model as clinically relevant as possible, and user friendly. In modern health care using patient reported outcome measures (PROMs), like the QoR-40 score, have become increasingly important. Combining PROMs with common available clinical variables makes the presented prediction model appealing for both clinicians and patients.

We first build a basic model using clinical variables. Next, to build the full model patient-reported measures were added to the basic model. The prognostic variables contributing the most to the model included the HADS, preoperative QoR-40 score and the duration of surgery. All used variables were previously described in the literature as variables having a predicting property for the quality of postoperative recovery^5,10–13^.

Patients’ demographic and clinical characteristic had a relatively minor impact in the model, whereas mental state and the duration of the surgical procedure were of prominent importance. The experience with previous surgery is only a small contributor to both models and the effect differs. Both confidence intervals are wide indicating the predictor is not very specific. They both cover the range from a negative to a positive effect on the outcome being the quality of postoperative recovery. We emphasize that this result could be due to statistically random fluctuation. We see the same effect with wide confidence intervals in the ASA classification when further observing the nomogram and tables. We found that an ASA classification of 2 was prognostic worse for the quality of recovery than having a score of 3. A possible explanation for this phenomenon is that patients with an ASA classification score of 3 are preoperatively already more dependent on health services whereas ASA classification score 2 patients are well controlled in their daily life. After surgery they become more care dependent, and this could be experienced more negatively by ASA 2 patients compared to ASA 3 patients.

The utility of a clinical prediction model is reflected by its performance measures. For patient counselling, model calibration is highly consequential as it may lead to harmful decisions^21^. Therefore, calibration is described as the Achilles heel of a prediction model^22^. A model with moderate calibration and less optimal other model performance measures may be preferred above a model with better model performance measures but poor calibration^22,23^. The presented model here shows moderate calibration on external validation, meaning that the risk predictions are accurate. We observed a better calibration for QoR-40 scores between 160 and 185. However, in patients at the borders of the normal range (below 160 and above 185) the model showed worse calibration. Both the developmental data set and the external validation data set contained very few outliers in these ranges. In all clinical studies performed with the QoR-40 scores the vast majority of patients scores between the 160 and 185 preoperatively and returns to this value after recovery from surgery^3,4,10^. In addition, as expected, the external validation showed a lower *R*^2^ than observed in the development stage. The different hospital setting, and time period are likely responsible for this.

Both models were corrected for optimism using bootstrapping, which shows the difference between apparent model performance and true model performance coming from the study sample derived population and the underlying population respectively. Due to the optimism correction, the *R*^2^ of the models were lower. The optimism-corrected *R*^2^ of 0.15 of the full model means that 15% of the postoperative QoR-40 score is explained by the prediction model. At the moment, there are no previous prediction models to compare and this model is a starting point to find out which model performance can be obtained. We recommend increasing the *R*^2^ before implementation of the model in clinical practice. This may be done by adding additional prognostic variables or finding other relevant strong variables and should be subjected to further studies with larger sample sizes. In the retrospective character of this study, we did not want to add too many prognostic variables for sample size considerations. Based on subject matter knowledge and a systematic literature review, we choose predefined prognostic variables that were easily accessible and applicable in daily clinical practice. It is also possible to select prognostic variables using statistical procedures. These strategies however have many drawbacks, especially when applied to small data sets, and is not recommended^17,24^. Due to repeated significance testing automated stepwise selection procedures tend to provide too extreme predictor effects and thereby an overfitting model^25^. However, there could be (yet unknown) variables that might increase the *R*^2^ of the current model. Recent studies (e.g. Enhanced Recovery After Surgery programs) indicated preoperative physical activity, nutrition status, minimal invasive surgery techniques or anaesthetic regimens and possible postoperative ICU requirement as possible prognostic variables of interest. Expanding the set of patients will allow to study these variables of interest to update our model^15^. In general it is recommended to update prediction models and not develop prediction models de novo^17^. Therefore, the prediction model presented here could well be used as framework for future updating.

We used the QoR-40 score to predict the quality of recovery after surgery. This questionnaire is easy in use, available and validated in multiple languages. However, all items included by the QoR-40 could possibly be not specific enough to describe a poor or good quality of recovery. Perhaps outcome of surgery and perioperative complications are factors that might be of interest to be included to measure this. Future research could therefore also be subjected to the possible need of a tool that anticipates on surgical outcome and perioperative complications too. In order to prevent loss of information in the model, we choose to analyse the QoR-40 score as a continuous outcome without dichotomizing the score as recommended^17^. We used the QoR-40 score in favour of the QoR-15 score because the QoR-40 has demonstrated superior validity and reliability and provides a more extensive evaluation of the postoperative recovery. The model does not predict direct (or early) postoperative recovery because the QoR-40 scores were assessed at POD 7 .

The quality of recovery or even broader ‘the outcome after anaesthesia or surgery’ is difficult to predict with a variety of possible prognostic variables. Future research should focus on selected patient centred outcomes like Moonesinghe and the StEP-COMPAC Group describe^26^. In a systematic review and three-step Delphi consensus process they established standardised endpoints for use in perioperative clinical trials. Their recommendations could be of value as an addition to the current model to predict the quality of recovery in patients. We did not have data on most of these endpoints in the randomized controlled trials that were used during the development of our model.

This study has several strengths and limitations. First, data from the studies used were derived from two blinded randomised controlled trials ensuring high quality data, however could probably cause selection bias by the inclusion criteria of both trials. The studies used included different surgical patients (major inpatient surgery and minor outpatient surgery) which allowed for the domain external validation. This is a very rigid form of external validation^27^. However, different prediction models for both separate patient categories could be imaginable. We choose these datasets because of the excellent quality of the data. Further external validation for inpatients is needed on geographical and temporal terms^28^. The calibration of the model suggests that the model will perform well in outpatients too. Calibration was less in the outliers. Second, the external validation took place in the same hospital and geographical area. The generalizability to non-academic hospitals remains to be tested. Both datasets explored the effect of benzodiazepine premedication on the quality of recovery. However, they used different benzodiazepines in their studies. Although we controlled for the benzodiazepine prescription, this could limit the external validity to other countries, cultures or even surgical categories. Nevertheless, our datasets resulted in moderate calibration meaning that the risk prediction is reliable in different surgical settings. Third, the QoR-40 has bounded outcomes between 40 and 200. We did not use a specific model for bounded outcomes. Fourth, we did not include frailty measurements in our prediction model because these data were not available from the datasets. Adding these could have improved the prediction model. Last, more advanced calculations for sample size calculations are available. We calculated the expected MMOE being equal to 1.12. Our sample size could be considered just to small based on our methods however we judge it to be sufficiently close to the recommended cut-off value in the paper to proceed with the model development^18^. Our model can be used for future sample size calculations.

## Conclusion

We developed and externally validated a prediction model that predicts the quality of recovery up to one week after surgery with easy-to-use prognostic variables. Calibration of the model in a different surgical setting was favourable, although external validation in other settings is still desired. Furthermore, increasing the explained variance by the model is needed before clinical implementation can take place. The presented model can well be used for future updating attempts in prediction studies.

### Supplementary Information

Supplementary Information.

## Data Availability

The datasets used and analysed during the current study are available from the corresponding author on reasonable request.
