# Supplementary material for: Development and external validation of a clinical prediction model for predicting quality of recovery up to 1 week after surgery
Source: Sci Rep. 2024 Jan 3;14:387. doi: 10.1038/s41598-023-50518-1 (PMC10764891; doi:10.1038/s41598-023-50518-1)
Supplement: Supplementary file 1 — Supplementary Information. [file 41598_2023_50518_MOESM1_ESM.docx]

**Supplementary Material**

**Appendix 1**

The predicted quality of recovery one week after surgery for the full model is given by: (145.88 + 1.35* (age/10) + -2.70*sex (male = 0, female = 1) + -0.55*previous surgery (no = 0, yes = 1) + 0.27*BMI + ASA (ASA1 = 0, ASA2 = -3.29, ASA3 = -2.86) + -2.76*(duration/60) + -0.89*HADS POD 0 + 0.17*QoR POD 0
